# Sexual selection on cuticular hydrocarbons in the Australian field cricket, *Teleogryllus oceanicus*

**DOI:** 10.1186/1471-2148-9-162

**Published:** 2009-07-13

**Authors:** Melissa L Thomas, Leigh W Simmons

**Affiliations:** 1 Centre for Evolutionary Biology, School of Animal Biology, The University of Western Australia, Perth, Australia

## Abstract

**Background:**

Females in a wide range of taxa have been shown to base their choice of mates on pheromone signals. However, little research has focussed specifically on the form and intensity of selection that mate choice imposes on the pheromone signal. Using multivariate selection analysis, we characterise directly the form and intensity of sexual selection acting on cuticular hydrocarbons, chemical compounds widely used in the selection of mates in insects. Using the Australian field cricket *Teleogryllus oceanicus *as a model organism, we use three measures of male attractiveness to estimate fitness; mating success, the duration of courtship required to elicit copulation, and subsequent spermatophore attachment duration.

**Results:**

We found that all three measures of male attractiveness generated sexual selection on male cuticular hydrocarbons, however there were differences in the form and intensity of selection among these three measures. Mating success was the only measure of attractiveness that imposed both univariate linear and quadratic selection on cuticular hydrocarbons. Although we found that all three attractiveness measures generated nonlinear selection, again only mating success was found to exert statistically significant stabilizing selection.

**Conclusion:**

This study shows that sexual selection plays an important role in the evolution of male cuticular hydrocarbon signals.

## Background

It is common in natural populations for individuals of one sex, usually the female, to prefer certain phenotypic trait values over others in their choice of mates. Female preferences for male sexual signals are responsible for a spectacular array of phenotypic diversity found in the natural world, driving the evolution of exaggerated traits such as colouration [1], conspicuous ornaments [2,3], and song [4,5]. Females have also been found to base their choice of mate on pheromone signals. Although less well studied, pheromone signals are subject to the same kinds of natural and sexual selective forces that shape visual and auditory signals [6]. However, our understanding of the processes driving the evolution of pheromones is significantly less well developed.

Cuticular hydrocarbons are chemical compounds found on the cuticle of most terrestrial arthropods. These compounds have been studied extensively for their role as signals in mate and species recognition, and ecology [7,8]. Cuticular hydrocarbons are highly sexually dimorphic in a range of species, with many of the compounds present in one sex but absent in the other, while shared compounds often differ quantitatively between the sexes [see [9] for review]. Such sexual dimorphism is expected to result from sex-specific selection. Despite the large number of species that display sexual dimorphism for cuticular hydrocarbons, to date, research on sexual selection acting on these compounds is limited, and primarily focuses on species in the genus *Drosophila*. Work in natural populations of *Drosophila serrata *indicates that cuticular hydrocarbons are sexually selected as a consequence of mate choice within populations, when measured on either lab-reared [10] or field-collected phenotypes [11,12]. Cuticular hydrocarbons have also been shown to evolve rapidly in *D. serrata*, when mate recognition is under selection [13]. However, aside from studies of *Drosophila *[[14], e.g. [15,16]], characterisation of the form and intensity of sexual selection acting on cuticular hydrocarbons is lacking.

Here we characterise the form and strength of sexual selection acting on male cuticular hydrocarbons through female mating preferences in the Australian field cricket *Teleogryllus oceanicus*. Although most interest in Orthopterans has focussed on auditory communication, increasing evidence suggests that cuticular hydrocarbons provide a significant additional communication channel. Sexual dimorphism of cuticular hydrocarbons has been found in at least five species of Orthoptera [9], and studies have shown that cuticular hydrocarbons are involved in mate recognition [17,18], mate choice [17], and pre-mating species isolation [19]. Twenty-two compounds have been identified from the cuticle of male *T. oceanicus *[20]. Some of these compounds show high levels of additive genetic variance while others do not. Studies of *Drosophila *have shown that sexual selection can erode genetic variance along the axes of variation in multivariate trait space that are under selection, and yet maintain considerable additive genetic variance along axes orthoganol to those under selection [11,21]. Thus, the patterns of heritable variation we see among individual *T. oceanicus *cuticular hydrocarbons might be explained by sexual selection acting on their relative abundances.

We use a multivariate approach to estimate how different hydrocarbon components on the cuticle of *T. oceanicus *combine to determine male attractiveness, and thus the form and intensity of sexual selection acting on the male cuticular hydrocarbon profile. Using a multivariate approach has the advantage of being able to describe how all traits interact to determine fitness. This is important because female *T. oceanicus *could base their choice of mate, either on the amount of one or more compounds independently, or on a particular combination of different male hydrocarbons.

## Methods

Animals for this experiment were the direct offspring from field collected females originating from a banana plantation in Carnarvon, Western Australia. All crickets were maintained in a constant temperature room (25°C), maintained on a 12:12 h light:dark cycle. They were supplied with water and fed cat chow ad libitum. Sexes were separated before the penultimate instar. Following the imaginal molt, experimental crickets were housed individually in boxes (7 cm × 7 cm × 5 cm). To ensure sexual receptivity, crickets were left to mature for 14 ± 3 days before being used in experiments.

### Mating trials

To quantify male attractiveness we measured both pre- and postcopulatory mating success when males were mated to five different females over five consecutive days. Prior to their use in experiments, females were provided with a male from the stock population for twelve hours. This is because, in some species of crickets, a female's choosiness increases after her first mating [22]. To minimize observer disturbance during mating trials, we conducted trials in a room dimly lit by red incandescent lights. We also placed each mating pair in a small plastic box (7 cm × 7 cm × 5 cm) that was then placed inside a larger plastic box (17 cm × 12 cm × 6 cm), which was lined with packing material on three sides to minimize the mating pairs exposure to other mating pairs under observation.

As a measure of precopulatory mating success we used (1) the duration of courtship required to persuade the female to mount, hereafter referred to as courtship duration, and (2) the number of successful matings (out of a possible five). A mating was scored as successful when a male successfully attached a spermatophore so that sperm had the opportunity to be transferred to the female. Therefore, courtships in which females failed to mount the male, or in which the male was unable to attach a spermatophore were scored as unsuccessful. The reciprocal of courtship duration was used as a measure of male success on the grounds that unattractive males should be expected to have to deliver greater amounts of courtship in order to persuade a female to mount. As a measure of postcopulatory mating success we used spermatophore attachment duration. For many cricket species, including *T. oceanicus*, increasing the duration of spermatophore attachment typically results in an increase in the number of sperm transferred [23-26]. So for females, the timing of spermatophore removal can be a form of postcopulatory choice that can bias paternity towards attractive males [23,24,27]. To provide females with adequate postcopulatory choice, we removed males from mating boxes immediately following copulation. This is because males aggressively oppose spermatophore removal by females during postcopulatory mate guarding [28,29]. Moreover, recent evidence in *T. commodus *suggest that when males are allowed to harass females during mate guarding, sexual selection is weaker [30].

### Cuticular hydrocarbon analysis

To quantify differences in cuticular hydrocarbon profiles, we immersed freshly freeze killed individual crickets in 5 ml of hexane for five minutes. We injected 1 μl of this sample into a gas chromatograph and mass spectrometer (Shimadzu GCMS QP2010) operating in the split mode, and fitted with a Stabilwax column of 30 m × 0.25 mm internal diameter using helium as a carrier gas. We optimized separation of the extract by using a column temperature profile in which the analysis began at a temperature of 50°C for 1 minute, and rose to 250°C for 20 minutes. The transfer line from the GC to the mass spectrometer was set at 250°C. We analysed washes derived from males that began courting all five females (92 males). We also analysed hexane blanks to control for potential contamination of samples.

For data analysis, peaks were labelled by peak number, which corresponded to their retention times (Table 1). Cuticular hydrocarbon profiles of each male consisted of the relative abundances (peak areas) of twenty-two individual compounds. This compositional dataset was transformed to logcontrasts (using peak 3 as the divisor), as described previously for *T. oceanicus *cuticular hydrocarbon data [9,20]. We performed a principal components analysis (PCA) on these log contrasts. The PCA provided a new set of standardized uncorrelated variables, ideal for calculating multivariate selection gradients [31]. Individual hydrocarbons were identified by their retention times and mass spectra (see Table 1).

**Table 1:** The principal component analysis of trait loading shows the correlations between the relative concentrations of cuticular hydrocarbon peaks and the six components extracted from the principal component analysis.

		Principle component					
		
Peak	Hydrocarbon	1	2	3	4	5	6
1	unresolved	0.230	0.351	-0.347	0.214	-0.366	-0.318
2	unresolved	0.495	-0.045	-0.192	-0.339	-0.002	-0.303
4	unresolved	0.156	-0.162	-0.184	**0.708**	0.276	-0.294
5	C_31:1_	-0.203	-0.030	-0.215	0.011	0.346	**0.630**
6	C_31:1_	-0.048	0.438	0.179	0.217	**-0.740**	0.128
7	C_31:1_	0.327	**0.649**	**0.593**	0.036	-0.066	0.001
8	C_31:2_	0.105	**0.845**	0.212	0.281	0.006	0.100
9	C_31:2_	0.586	**0.708**	-0.177	-0.103	0.123	0.074
10	C_31:2_	0.532	**0.762**	-0.102	-0.199	0.172	0.029
11	C_31:2_	-0.234	-0.144	**0.584**	0.090	0.091	-0.083
12	C_33_	0.402	**0.750**	0.071	-0.083	0.441	-0.137
13	unresolved	0.458	-0.538	0.468	-0.097	0.076	-0.187
14	C_33:1_	0.400	0.070	-0.419	0.445	-0.134	0.112
15	C_33:1_	0.284	-0.150	-0.314	**0.706**	-0.008	-0.034
16	C_33:1_	**0.795**	-0.435	-0.149	-0.044	0.186	-0.125
17	C_33:1_	0.165	-0.146	**0.719**	0.453	-0.026	0.075
18	C_33:2_	**0.796**	-0.190	-0.190	-0.267	-0.312	0.171
19	C_33:2_	**0.809**	-0.411	0.046	-0.126	-0.279	0.190
20	C_33:2_	**0.880**	-0.229	0.236	-0.113	-0.070	0.072
21	C_33:2_	**0.721**	-0.118	0.248	0.199	0.204	0.051
22	C_35:2_	0.326	-0.224	-0.034	0.297	0.138	0.355

Peaks that contribute significant amounts to the principal components are in bold.

### Characterizing sexual selection

To characterise the form of sexual selection imposed on cuticular hydrocarbons we used separate multiple regressions to estimate the vector of linear selection gradients (*β*), and the matrix of quadratic and correlational selection gradients (*γ*) for each measure of male attractiveness [31]. Before analysis, it was necessary to transform two of our attractiveness measures, courtship duration and spermatophore attachment duration, using natural logarithms as both were skewed. We also scaled each of our attractiveness measurements to relative fitness measures by dividing each datum by the mean.

To determine the significance of linear and nonlinear sexual selection for each measure of male attractiveness, we assessed the fit of the respective models. To evaluate if univariate linear selection was occurring, we used the overall significance of the regression model incorporating only the linear (*β*) terms. We also calculated univariate nonlinear selection gradients (*γ*) by incorporating the quadratic and correlational terms. However, univariate analyses of complex traits like cuticular hydrocarbon blends can significantly underestimate nonlinear selection operating in multivariate trait space [32]. We therefore used response surface methodology [33], implemented in JMP^®^7, to test for the significance of nonlinear selection. Because we used response surface methodology, there was no need to double the quadratic coefficients [34]. This method results in a canonical analysis of the matrix of nonlinear selection gradients. The canonical analysis allows an interpretation of both concave and convex selection to be made on combinations of traits that describe the greatest amount of nonlinear variation on fitness surfaces [33-35]. The resulting eigenvectors (*m*_*i*_) from the canonical analysis denote the major axes that constitute the M matrix. These indicate how the original traits, or principal components, contribute to the major axes of the response surface. To evaluate the significance of nonlinear selection, new variables were created from the eigenvectors of *γ *and a second quadratic regression was performed using these new variables [35].

To visualise the different nonlinear selection patterns, we compared the fitness surfaces comprising the major axes of the separate canonical rotations using the bicubic spline smoothing procedure in the program Statistica 6.0. The stiffness parameter was automatically set at 0.25 for all figures. Visualisation of these splines suggested that female choice generated both stabilizing and disruptive selection on all three measures of attractiveness. Documenting a significant quadratic term is not sufficient to conclude that a fitness peak occurs at the extremes (disruptive selection) or at intermediate (stabilizing selection) trait values [36]. We therefore used the statistical procedures originally outlined by Mitchell-Olds and Shaw [36] to test for significant stabilizing and disruptive selection. Briefly, this method tests for a non-intermediate optimum by constraining the fitness optimum at either the maximum or minimum of the phenotypic distribution. We then test whether this new constrained phenotypic distribution produces a significantly worse fit than the unconstrained (original) model, using standard techniques for testing a general linear hypothesis. Further details outlining implementation of this procedure can be found in Chenoweth et al. [37].

## Results

Consistent with our previous studies of male *T. oceanicus*, we were able to identify 22 peaks that ranged in chain length from 31 to 35 carbons. Principal component (PC) analysis returned 21 components. We used only those components where the eigenvalue was greater than 1 [38] in our selection analyses. Six components had an eigenvalue greater than 1 and collectively explained 75% of the variance in cuticular hydrocarbon blend. The percentage of variance explained was 24.40, 19.01, 10.70, 9.50, 6.81 and 4.78 for components 1–6 respectively. The principal component analysis matrix of trait loading (Table 1) shows how the original peaks contribute to each PC. Correlations greater than 0.7 times the largest correlation were considered to have contributed significantly to the PC [39]. PC1 was most strongly loaded by peaks corresponding to C_33 _alkenes, whereas PC2 was most strongly loaded by peaks corresponding to C_31 _alkenes. The remaining PCs can be interpreted as representing relative abundances of a range of hydrocarbons (Table 1).

We calculated the repeatability of a male's attractiveness across the five females each male was offered for the variables courtship duration and spermatophore attachment duration. Repeatabilities were based on the variance components derived from an analysis of variance (ANOVA), with equal sample sizes (courtship duration) and unequal sample sizes (spermatophore attachment) [40]. The repeatability estimates for courtship duration and spermatophore attachment duration were 0.13 (F_91,368 _= 1.715, p < 0.001) and 0.05 (F_91,319 _= 1.02, p = 0.436) respectively. These low repeatabilities could be attributed to differences in stochastic encounters with females early and late in the mating trials. For example, deposition of hydrocarbons from a male's previous mating partner could affect mating behaviour of his subsequent partners. Indeed a recent publication demonstrated strong effects of deposited male hydrocarbons on male ejaculate expenditure [17]. To determine if this is the case, we examined the difference between selection acting on male hydrocarbons when they are mated to their first female compared with when they are mated with their last female. Using methods outlined in [16], we found no significant difference in the fitness surfaces between the two groups for linear (F = 0.575' df = 6,170, p = 0.750), quadratic (F = 0.232, df = 6,158, p = 0.966) or correlational (F = 0.663, df 15, 128, p = 0.816) selection (see Additional file 1). In subsequent analyses we therefore use the average of each trait measured across the five females with which males copulated, as a measure of attractiveness for each male.

Pearsons correlations showed that mating success was positively correlated with both the reciprocal of courtship duration (r = 0.418, n = 92, p < 0.001), and with spermatophore attachment duration (r = 0.517, n = 92, p < 0.001). Thus, males with higher overall mating success needed to perform less courtship per mating, and females retained their spermatophores for longer than unsuccessful males. However, relative spermatophore attachment and relative courtship duration were not strongly correlated (r = 0.170, n = 92, p = 0.105), suggesting that they may represent relatively independent contributions to male reproductive success.

All measures of male attractiveness generated significant sexual selection on male cuticular hydrocarbons. The percentage of variance explained by these models were 54%, 29% and 31% for mating success, courtship duration and spermatophore attachment duration respectively. The standardized univariate linear, quadratic, and correlational selection gradients for each measure of male attractiveness are presented in Tables 2, 3, and 4. When relative mating success was used as the response variable, we found significant linear selection on PC2 (Table 2). We also found significant correlational selection between this component and PC3 (Table 2). Although, we found no significant linear, quadratic or correlational selection on cuticular hydrocarbons arising from courtship duration, we did find correlational selection between PC1 and 4 for the attractiveness measure, spermatophore attachment (Tables 3 and 4). Moreover, we found significant multivariate nonlinear selection acting on male hydrocarbon profiles via all three of our attractiveness measures (Tables 5, 6 and 7).

**Table 2:** The vector of standardized linear selection gradients (β), and the matrix (γ) of standardized quadratic and correlational selection gradients for the six principal components with eigenvalues greater than one.

Relative mating success							
	β	PC1	PC2	PC3	PC4	PC5	PC6

PC1	0.004	-0.001	0.001	**0.027	0.002	-0.006	0.006
PC2	**0.018	.	0.009	*-0.022	0.012	-0.019	-0.009
PC3	0.048	.	.	0.002	0.009	*-0.037	*-0.049
PC4	-0.013	.	.	.	0.012	-0.003	0.033
PC5	-0.020	.	.	.	.	-0.022	0.009
PC6	-0.025	.	.	.	.	.	**-0.054

* P < 0.05, ** P < 0.01, *** P < 0.001

The response variable is relative mating success. Linear and nonlinear selection gradients were estimated in separate regressions.

**Table 3:** The vector of standardized linear selection gradients (β), and the matrix (γ) of standardized quadratic and correlational selection gradients for the six principal components with eigenvalues greater than one.

Inverse relative courtship duration							
	β	PC1	PC2	PC3	PC4	PC5	PC6

PC1	0.011	-0.006	0.001	0.009	0.001	0.008	-0.011
PC2	0.019	.	-0.002	-0.006	0.012	0.003	-0.009
PC3	0.018	.	.	-0.010	-0.012	-0.015	-0.043
PC4	-0.018	.	.	.	0.003	0.021	0.011
PC5	-0.047	.	.	.	.	0.008	0.035
PC6	-0.038	.	.	.	.	.	-0.027

No significant values

The response variable is inverse relative courtship duration. Linear and nonlinear selection gradients were estimated in separate regressions.

**Table 4:** The vector of standardized linear selection gradients (β), and the matrix (γ) of standardized quadratic and correlational selection gradients for the six principal components with eigenvalues greater than one.

Relative spermatophore attachment duration							
	β	PC1	PC2	PC3	PC4	PC5	PC6

PC1	0.005	0.008	-0.001	0.016	*0.019	-0.009	0.018
PC2	<0.001	.	0.013	-0.011	-0.004	-0.012	-0.026
PC3	-0.016	.	.	0.009	-0.004	-0.037	-0.064
PC4	-0.067	.	.	.	-0.011	0.005	-0.021
PC5	-0.025	.	.	.	.	-0.023	-0.007
PC6	-0.034	.	.	.	.	.	-0.049

* P < 0.05

The response variable is relative spermataphore attachment duration. Linear and nonlinear selection gradients were estimated in separate regressions.

**Table 5:** M matrix of eigenvectors from the canonical analysis of γ for the 6 principal components. θ is the multivariate linear selection.

Relative mating success								
*m*_*i*_	θ	λ_i_	PC1	PC2	PC3	PC4	PC5	PC6

*m*_1_	*0.029	***0.027	0.383	-0.288	0.784	-0.110	-0.286	-0.249
*m*_2_	0.017	***0.019	0.189	0.500	0.151	0.796	-0.223	0.092
*m*_3_	**-0.038	*0.009	-0.027	-0.766	-0.025	0.540	0.264	0.224
*m*_4_	-0.012	*-0.006	0.895	0.025	-0.300	-0.127	0.236	0.189
*m*_5_	0.012	***-0.033	-0.052	0.252	0.358	0.057	0.859	-0.255
*m*_6_	-0.009	***-0.069	-0.111	0.128	0.379	-0.208	0.074	0.882

Note: The eigenvalue (λ_i_) of each eigenvector (*m*_*i*_) is given in the third column.

* P < 0.05, ** P < 0.01, *** P < 0.001

The response variable is relative mating success.

**Table 6:** M matrix of eigenvectors from the canonical analysis of γ for the 6 principal components and weight.

Inverse relative courtship duration								
*m*_*i*_	θ	λ_i_	PC1	PC2	PC3	PC4	PC5	PC6

*m*_1_	*-0.061	**0.030	-0.028	0.107	-0.415	0.458	0.669	0.398
*m*_2_	0.029	0.004	0.271	0.715	0.233	0.505	-0.124	-0.302
*m*_3_	-0.005	<-0.001	0.705	-0.259	0.394	-0.148	0.493	-0.127
*m*_4_	0.008	-0.008	0.033	0.624	-0.191	-0.717	0.235	0.065
*m*_5_	-0.009	**-0.014	-0.649	0.062	0.598	-0.007	0.437	-0.165
*m*_6_	-0.009	***-0.045	0.088	0.131	0.473	-0.004	-0.219	0.839

θ is the multivariate linear selection.

Note: The eigenvalue (λ_i_) of each eigenvector (*m*_*i*_) is given in the third column.

** P < 0.01, *** P < 0.001

The response variable is relative courtship duration.

**Table 7:** M matrix of eigenvectors from the canonical analysis of γ for the 6 principal components and weight.

Relative spermatophore attachment duration								
*m*_*i*_	θ	λ_i_	PC1	PC2	PC3	PC4	PC5	PC6

*m*_1_	<0.001	**0.030	0.281	0.061	0.845	0.084	-0.307	-0.318
*m*_2_	0.005	**0.017	-0.359	0.903	-0.032	-0.117	-0.069	-0.192
*m*_3_	-0.006	*0.010	0.813	0.382	-0.280	0.309	-0.009	0.138
*m*_4_	**-0.019	-0.009	-0.255	-0.029	0.006	0.857	0.327	-0.304
*m*_5_	0.002	*-0.029	0.212	0.066	0.192	-0.343	0.865	-0.218
*m*_6_	-0.018	***-0.072	-0.147	0.174	0.411	0.175	0.213	0.838

θ is the multivariate linear selection.

Note: The eigenvalue (λ_i_) of each eigenvector (*m*_*i*_) is given in the third column.

* P < 0.05, ** P < 0.01, *** P < 0.001

The response variable is relative spermatophore attachment duration.

**Figure 1:**
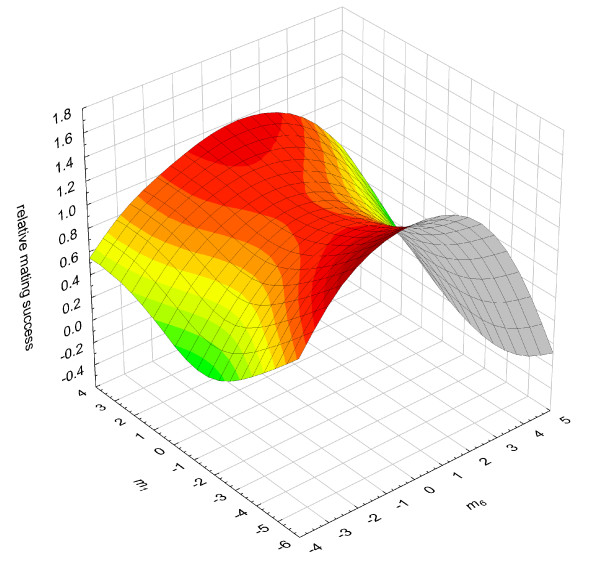
**The fitness surface of the major canonical axes *m*_1 _and *m*_6 _of the attractiveness measure, relative mating status**. The two axes represent the eigenvectors with the strongest positive (*m*_1_) and negative (*m*_6_) eigenvalues. The surface is shaded in colours where red corresponds to the greatest and green the lowest values on the z axis.

To visualise the different nonlinear selection patterns imposed by each variable, we compared fitness surfaces comprising the major axes of the separate canonical rotations (Tables 5, 6, and 7). The canonical analysis shows the eigenstructure, which is useful for identifying and describing the shape and orientation of the curvature of the nonlinear response surface. For the attractiveness measure, relative mating success, all six axes displayed nonlinear selection as indicated by the significant eigenvalues, which represent both concave (*m*_1–3_) and convex (*m*_4–6_) selection (Table 5). When plotting the fitness surface for the two major canonicals which represent the eigenvectors with the strongest positive (*m*_1_) and negative (*m*_6_) eigenvalues for this attractiveness measure, the surface is a peak along the *m*_6 _axis, suggestive of stabilizing selection (Figure 1). Formal statistical testing for stabilizing selection (a peak in fitness at intermediate values of *m*_6_), showed that models constrained to have their maxima at either the minimum (t_89 _= 5.17, p < 0.001) or maximum (t_89 _= -4.54, p < 0.001) of the phenotypic distribution produced significantly worse fits than the unconstrained model. This indicates an intermediate optimum fitness value for this measure of male attractiveness, and thus significant stabilizing selection on male cuticular hydrocarbons. Along the *m*_1 _axis, the surface is a saddle with peaks at the extremes of the *m*_1 _axis, suggestive of disruptive selection (Figure 1). However, when a formal analysis for disruptive selection was conducted, we found that models constrained to have peaks only at their minimum (t_89 _= 0.84, p = 0.401) or maximum (t_89 _= 0.72, p = 0.474) of the phenotypic distribution, did not produce significantly worse fits than the unconstrained model, indicative of no significant disruptive selection.

**Figure 2:**
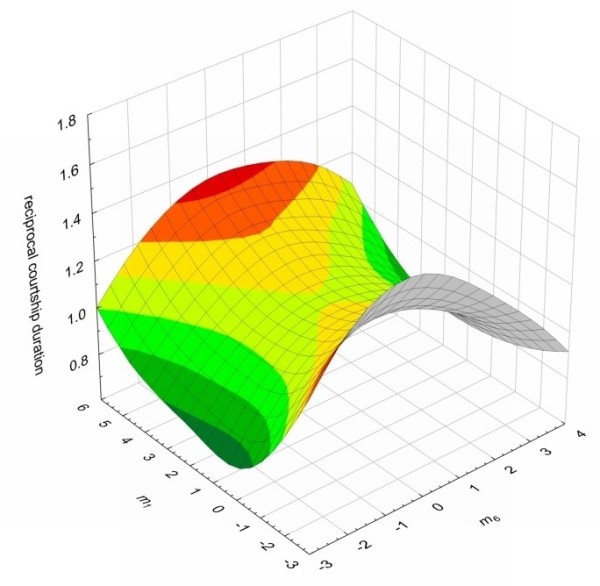
**The fitness surface of the two major canonical axes *m*_1 _and *m*_6 _of the courtship duration attractiveness measure**. The two axes represent the eigenvectors with both the strongest nonlinear selection (highest eigenvalues) and the strongest positive (*m*_1_) and negative (*m*_6_) eigenvalues. The surface is shaded in colours where red corresponds to the greatest and green the lowest values on the z axis.

**Figure 3:**
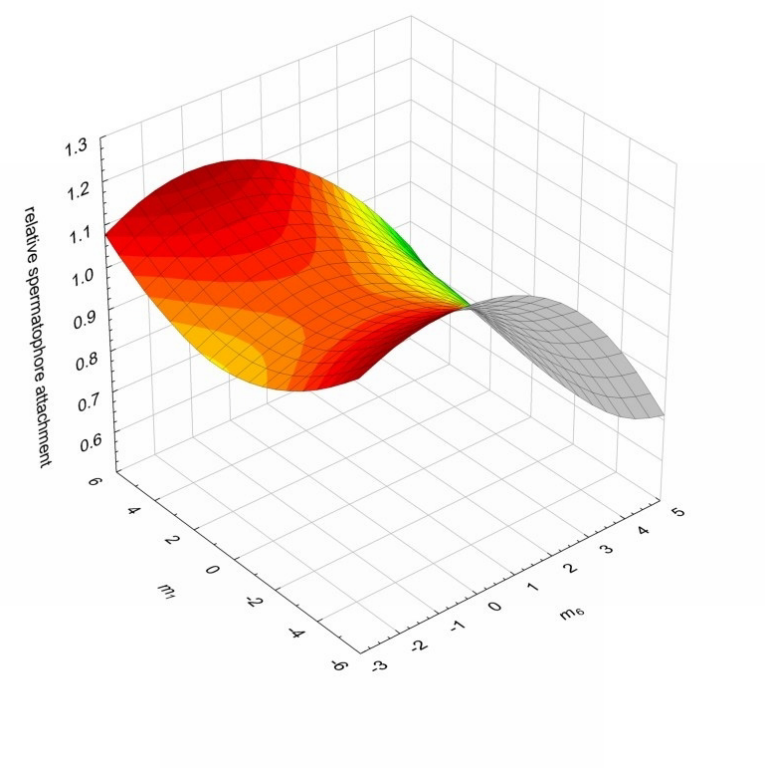
**The fitness surface of the two major canonical axes *m*_1 _and *m*_6 _of the spermatophore attachment attractiveness measure**. The two axes represent the eigenvectors with both the strongest nonlinear selection (highest eigenvalues) and the strongest positive (*m*_1_) and negative (*m*_6_) eigenvalues. The surface is shaded in colours where red corresponds to the greatest and green the lowest values on the z axis.

Similar patterns were also found for the remaining two attractiveness measures, relative courtship duration and spermatophore attachment. For both of these measures of attractiveness the surfaces were again peaks along the *m*_6 _axes, suggestive of stabilizing selection (Figures 2 &3). However, the trend towards stabilizing selection at an intermediate value of *m*_6 _was not statistically significant. In the case of relative courtship duration, a fitness peak constrained to be at the maximum phenotypic value for *m*_6 _did not quite produce a significantly worse fit (minimum, t_89 _= 2.50, p = 0.014; maximum, t_89 _= -1.76, p = 0.081), while in the case of relative spermatophore attachment duration, a fitness peak constrained to be at the minimum phenotypic value for *m*_6 _did not quite produce a significantly worse fit (minimum, t_89 _= 1.81, p = 0.074; maximum, t_89 _= -2.67, p = 0.009). When plotting the fitness surface for the major canonical which represents the eigenvectors with the strongest positive (*m*_1_) eigenvalue, the surfaces were saddles with peaks at the extremes of the *m*_1 _axis for both courtship duration and spermatophore attachment, again suggestive of disruptive selection along these axes (Figure 2 &3). However, we found no statistically significant disruptive selection along these axes for either attractiveness measure (Relative courtship duration; minimum (t_89 _= -2.37, p = 0.020), maximum (t_89 _= 1.36, p = 0.178); Relative spermatophore attachment; minimum (t_89 _= 0.73, p = 0.466), maximum (t_89 _= -1.35, p = 0.180)).

## Discussion

We have shown that sexual selection acts on the cuticular hydrocarbon profile of male *T. oceanicus*. Although we found that all three measures of male attractiveness generated sexual selection on male cuticular hydrocarbons, there were differences in the form and intensity of selection among these three measures. For example, relative mating success was the only measure of attractiveness that generated both univariate linear and quadratic selection. Similarly, although we found nonlinear selection generated by all three attractiveness measures, only mating success was found to exert statistically significant stabilizing selection.

Stabilizing selection is convex nonlinear selection in which the optimum phenotype is at an intermediate point in the range of phenotypes in the population [31]. In theory, signals that are required to be highly species specific, such as those used in mate choice, could be under strong stabilizing or directional selection. Stabilizing selection will enhance selection against small changes in the signal, whereas directional selection will favour more extreme male variants. Although we found significant convex nonlinear selection acting via all three attractiveness traits along the m6 axes of hydrocarbon variation (Figures 1, 2 and 3), only mating success was found to exert statistically significant stabilizing selection. Nonetheless, this same pattern of stabilizing selection was also present for the remaining two attractiveness traits along this axis. This is perhaps not surprising given the correlation between mating success and both courtship duration and spermatophore attachment duration. For all of our attractiveness measures, the m6 axis was most strongly associated with positive values of PC3 and PC6. These PCs account for variation in three hydrocarbons (peaks 7, 11 and 17) and one hydrocarbon (peak 5) respectively. These hydrocarbons have previously been shown to be sexually dimorphic, being more abundant in males than females [[9] note that peaks 5, 7, 11 and 17 correspond to peaks 7, 9, 13 and 19 respectively, in this paper]. Strong stabilizing selection should erode additive genetic variance in the traits under selection. Indeed, previous work with *Drosphila* has found that additive genetic variation in cuticular hydrocarbons has been eroded along the major axis of selection [11,21]. All three peaks that contribute to PC3 and the one peak that contributes to PC6 have significant levels of additive genetic variation, with CVAs of 6.7, 11.8 and 31.8 for peaks 7, 11, and 17 respectively, and 21.5 for peak 5 [20]. It remains to be established whether there remains significant additive genetic variation along the major axes of multivariate selection.

Disruptive selection is concave nonlinear selection in which the optimum phenotype is at the extremes in the range of phenotypes in the population. Disruptive selection has been implicated in the maintenance of polymorphism in traits generally related to fitness [32,41,42]. Although we found concave selection along the m1 axes imposed by all of our attractiveness measures (Figures 1, 2 and 3), we found no statistical support for disruptive selection along this axis for any of our attractiveness measurements. This suggests that female *T. oceanicus* do not prefer rare cuticular hydrocarbons, but rather female mate choice in this species appears to be driving male cuticular hydrocarbons to a single most attractive peak.

Our estimate of the intensity of nonlinear sexual selection on cuticular hydrocarbons generated by spermatophore attachment duration was considerably lower than the selection imposed by spermatophore attachment duration on male courtship song in *T. commodus *[30]. The largest absolute eigenvalue in our analysis (0.072) was an order of magnitude lower than the equivalent value reported for *T. commodus *(0.860). Moreover, the work with *T. commodus *revealed that when males were allowed to guard females after mating, the opportunity for selection was greatly reduced, the form of selection changed, and sexual selection was significantly weakened. Thus, although we found weak postcopulatory sexual selection to act via spermatophore attachment duration in the absence of male guarding, the findings for *T. commodus *suggest that this selection is likely to be even weaker when male *T. oceanicus *guard their mates after copulation.

The results of our repeatability analysis further suggest that precopulatory sexual selection on cuticular hydrocarbon profiles is of greater significance in *T. oceanicus *than postcopulatory sexual selection via spermatophore removal; the repeatability of a male's courtship duration across multiple females was significant, whereas the repeatability of spermatophore attachment duration was not. This is somewhat consistent with work on the cricket *Acheta domesticus*. In *A. domesticus*, the timing of spermatophore removal by females is determined, in part, by the female's own genotype, independent of the quality of her mate [43]. It seems unlikely that the attractiveness of a female's previous mate would influence our repeatability results, since postcopulatory mate choice is not influenced by the attractiveness of a female's previous mate in other cricket species [27,44].

The difference in the form and intensity of selection acting via spermatophore attachment in *T. oceanicus *and *T. commodus*, and the considerably lower intensity of nonlinear sexual selection compared with our measures of precopulatory sexual selection, could be due to the effect, or lack thereof, of sperm numbers on the fertilization success of male *T. oceanicus*. In general, increased spermatophore attachment duration is known to increase the amount of sperm transferred to females [24-26], and this is also the case in *T. oceanicus *[26]. However, spermatophore attachment duration does not appear to strongly influence paternity success in *T. oceanicus*, primarily because sperm numbers *per se*, have no influence on the fertilization success of males when under sperm competition [26]. Rather, paternity success of *T. oceanicus *is determined by the proportion of live sperm in a male's ejaculate [45]. Although not yet examined, it is possible that sperm numbers may influence paternity success of *T. commodus*, enabling greater opportunities for effective female choice via spermatophore attachment duration in this species [30].

Although we have clearly shown that sexual selection acts on cuticular hydrocarbons, there remains a large proportion of variation in male fitness that cannot be explained by cuticular hydrocarbon profiles. It is therefore unlikely that females base their mate choice solely on these chemical cues. Acoustic signals produced by many insects, birds, and anuran amphibians are among the best-known examples of sexual signals used by males to attract mates. Male *T. oceanicus *produce a complex courtship song consisting of two elements, the short chirp followed by a prolonged trill, and females are known to prefer courtship songs with an overall higher duty cycle and sustained duration of trill [46]. Moreover, stabilizing sexual selection has been shown to act on the courtship song structure of a closely related species, *T. commodus *[47]. Since we did not control for male courtship song in this study, it seems likely that a proportion of the remaining variation in male fitness could be explained by differences in courtship songs between males. Furthermore, male *T. oceanicus *also produce a long-range calling song, consisting of a long chirp and a series of short chirps, which serves to attract females for courtship. Females prefer calling songs with high proportions of long chirp elements [48-50]. We have only examined selection acting on cuticular hydrocarbons during the short range contact phase of courtship. If the attractiveness of male calling song were positively associated with the attractiveness of the two short range signals, courtship song and cuticular hydrocarbon profile, total selection on cuticular hydrocarbons might be stronger. An interesting avenue for future research would be to explore the interrelationship between song and chemicals in inter-sexual communication.

Relatively few empirical studies have investigated the combined effects of multiple signals on female choice [e.g. [51,52] and [53]], and the trade-offs that may constrain the simultaneous expression of signals relevant to sexual selection [54]. A complete understanding of the evolution of male sexual signals will require the determination of total sexual selection operating over different episodes of female choice, and male contest competition [49].

## Conclusion

Our study provides one of only a few studies that formally characterises the form and intensity of sexual selection acting on cuticular hydrocarbons. Although all three measures of attractiveness used in this study were found to exert sexual selection on cuticular hydrocarbons, there were differences in both the form and intensity of selection among these measures. Relative mating success was the only measure of attractiveness that generated both univariate linear and quadratic selection, in addition to statistically significant multivariate stabilizing selection. The presence of stabilizing selection suggests that female mate choice, in this species, appears to be driving male cuticular hydrocarbons to a single most attractive peak. Although a multivariate stabilizing peak was found in this system, a recent review of the few multivariate fitness surfaces available, suggests that stabilizing selection may in fact be uncommon [49]. The presence of multivariate stabilizing selection, therefore, remains an important but relatively unexplored aspect of sexual selection.

## Authors' contributions

MLT carried out the behavioural component of this manuscript. Both authors jointly conceived the study, helped to draft, and approved the final manuscript.

## Supplementary Material

Additional file 1 **Table A1 and A2**. Contrast in multivariate selection acting on previously unmated males, and males mated four times previously. Click here for file
