# Supplementary material for: Sexual selection on cuticular hydrocarbons in the Australian field cricket, Teleogryllus oceanicus
Source: BMC Evol Biol. 2009 Jul 13;9:162. doi: 10.1186/1471-2148-9-162 (PMC2728729; doi:10.1186/1471-2148-9-162)
Supplement: Supplementary file 1 [file 1471-2148-9-162-S1.doc]

Table A1. M matrix of eigenvectors from the canonical analysis of γ for the 6 principle components. The response variable is relative mating success when a male was previously unmated. θ is the multivariate linear selection.

m_i	θ	λ_i	PC1	PC2	PC3	PC4	PC5	PC6
m_1	-0.004	**0.044	-0.109	0.359	-0.089	0.828	-0.207	0.350
m_2	-0.044	*0.027	-0.193	0.248	-0.244	-0.089	0.849	0.336
m_3	-0.002	0.010	-0.146	-0.795	0.353	0.339	0.253	0.209
m_4	-0.001	0.003	0.925	0.075	0.233	0.063	0.174	0.224
m_5	***0.085	*-0.022	-0.265	0.398	0.862	-0.146	0.022	0.086
m_6	**0.097	***-0.083	0.063	0.115	0.107	0.408	0.376	-0.814

Note: The eigenvalue (λ_i) of each eigenvector (m_i) is given in the third column.

* P < 0.05, ** P < 0.01, *** P < 0.001

Table A2. M matrix of eigenvectors from the canonical analysis of γ for the 6 principle components. The response variable is relative mating success when a male had been mated four times previously. θ is the multivariate linear selection.

m_i	θ	λ_i	PC1	PC2	PC3	PC4	PC5	PC6
m_1	0.03	***0.056	-0.109	0.359	-0.089	0.828	-0.207	0.350
m_2	0.011	*0.029	-0.193	0.248	-0.244	-0.089	0.849	0.336
m_3	-0.001	0.016	-0.146	-0.795	0.353	0.339	0.253	0.209
m_4	0.009	-0.000	0.925	0.075	0.233	0.063	0.174	0.224
m_5	0.002	*-0.040	-0.265	0.398	0.862	-0.146	0.022	0.086
m_6	0.028	***-0.082	0.063	0.115	0.107	0.408	0.376	-0.814

Note: The eigenvalue (λ_i) of each eigenvector (m_i) is given in the third column.

* P < 0.05, ** P < 0.01, *** P < 0.001
